# Editorial: Current perspectives in the diagnosis of parathyroid disease, volume II

**DOI:** 10.3389/fendo.2026.1901912

**Published:** 2026-06-16

**Authors:** Takahisa Hiramitsu, Jong Ju Jeong

**Affiliations:** 1 Department of Transplant and Endocrine Surgery, Japanese Red Cross Aichi Medical Center Nagoya Daini Hospital, Nagoya, Aichi, Japan; 2 Division of Thyroid-Endocrine Surgery, Department of Surgery, Severance Hospital, Yonsei University College of Medicine, Seoul, Republic of Korea

**Keywords:** parathyroid hormone, hyperparathyroidism, parathyroid disease, parathyroid gland, parathyroidectomy

Parathyroid diseases encompass a diverse and complex range of endocrine disorders, ranging from asymptomatic biochemical abnormalities to aggressive conditions associated with severe systemic complications. Recent advances in biochemical assays and imaging modalities have substantially improved the detection of parathyroid disorders. Nevertheless, significant diagnostic challenges remain, including the accurate localization of lesions, reliable risk stratification, and the integration of diagnostic data into individualized clinical decision-making.

The second volume of the Research Topic *“Current perspectives in the diagnosis of parathyroid disease”* presents a collection of original research articles and comprehensive reviews that collectively demonstrate the evolving concept of diagnosis in parathyroid disease. Rather than focusing solely on biochemical confirmation or anatomical localization, this volume highlights a broader and more integrative diagnostic framework that incorporates biological heterogeneity, prognostic assessment, and emerging technological approaches.

In patients with primary hyperparathyroidism (PHPT), treatment decisions have traditionally been guided by biochemical profiles and symptomatic presentations. However, these conventional approaches cannot detect subclinical organ damage or predict disease progression. A previous review by Zhu et al. provided a comprehensive synthesis of emerging multilevel biomarkers for PHPT, including genetic and epigenetic alterations, non-coding RNAs, metabolic profiles, inflammatory markers, and advanced imaging features. The use of such dynamic biomarker profiling can facilitate precise risk stratification and support individualized treatment strategies. Furthermore, by proposing an integrated framework that combines dynamic biomarker assessment with multidisciplinary team-based decision-making, personalized surgical planning and long-term surveillance can be facilitated. This study underscores a significant conceptual shift from traditional uniform approaches toward personalized interventions and long-term surveillance.

In PHPT, preoperative localization of the pathological parathyroid glands (PTGs) remains a persistent challenge in minimally invasive parathyroidectomy (PTx). Kang et al. investigated the diagnostic performance of ^18^F-fluorocholine positron emission tomography-computed tomography (FCH PET/CT) and ^99m^Tc-sestamibi single-photon emission computed tomography/CT (MIBI SPECT/CT) by comparing preoperative imaging results with intraoperative localization. The sensitivity and positive predictive values were 74.4% and 86.1%, respectively, for MIBI SPECT/CT, and 97.5% and 91.5%, respectively, for FCH PET/CT. These findings indicate the superior performance of FCH PET/CT. Additionally, FCH PET/CT was associated with higher postoperative biochemical cure rates compared with MIBI SPECT/CT. Importantly, the study highlights that imaging accuracy is influenced by the preoperative functional activity and histologic composition of the PTGs.

In secondary hyperparathyroidism (SHPT), intact parathyroid hormone (PTH) and calcium levels have traditionally guided the assessment of treatment success following PTx. However, Feng et al. focused on the management of hyperphosphatemia. They developed and validated a predictive scoring model to estimate the reduction in serum phosphorus after total PTx. Preoperative levels of alkaline phosphatase, intact PTH, and free triiodothyronine were identified as independent factors influencing postoperative phosphorus decline. By incorporating these factors, along with preoperative serum phosphorus and the presence of bone pain—which act as inhibitory factors for phosphorus reduction—a predictive scoring model was established. The model’s utility was confirmed in a validation cohort. This study contributes to the early identification, prevention, and management of elevated phosphorus levels in patients undergoing total TPTx.

Gheorghe-Milea et al. conducted a systematic review evaluating inflammatory markers in PHPT. The review revealed that pro-inflammatory markers, including tumor necrosis factor-α, interleukin (IL)-6, high sensitivity C-reactive protein (Hs-CRP), and fibrinogen, were elevated in patients with PHPT. Additionally, PTH levels were found to correlate with IL-6, CRP, and white blood cell count. However, the effects of PTx on these inflammatory parameters remain controversial. These findings suggest that chronic inflammation may contribute to the multisystemic complications of PHPT, including cardiovascular and skeletal morbidities. From a diagnostic perspective, inflammatory biomarkers may serve as complementary indicators of disease activity or complication risk, particularly in asymptomatic patients or those deemed unsuitable for surgery.

Liu et al. developed a predictive model to differentiate parathyroid carcinoma (PC) and atypical parathyroid tumors (APT) from parathyroid adenoma (PA). Multiple logistic regression analysis identified intact PTH levels, tumor shape, and the relationship with the thyroid capsule on ultrasonography as independent predictive factors for APT/PC. A nomogram incorporating these factors was subsequently constructed. The high diagnostic accuracy observed in both training and validation cohorts highlights the value of integrating imaging characteristics with intact PTH levels. This approach provides a practical and accessible diagnostic tool that may enhance preoperative decision-making in routine clinical practice.

Haq et al. demonstrated the feasibility of generating patient-specific virtual three-dimensional anatomical models derived from four-dimensional CT imaging for minimally invasive PTx. These models reduce surgical workload, enhance spatial understanding, and improve patient education regarding disease anatomy and surgical planning. Such technological advances can improve diagnostic accuracy, surgical planning, and communication.

Zhao et al. demonstrated the diagnostic utility of the combined calcium–chloride/phosphorus ratio (([Ca]×[Cl])/P) and alkaline phosphatase (ALP) for PHPT. Patients with hypercalcemic PHPT showed significantly higher levels of PTH, ALP, Ca/P, Cl/P, and ([Ca]×[Cl])/P compared to those with normocalcemic PHPT and healthy controls. Furthermore, the combined marker of the ([Ca]×[Cl])/P ratio and ALP was useful for diagnosing and differentiating hypercalcemic from normocalcemic PHPT and showed moderate utility in distinguishing parathyroid adenoma from hyperplasia. The authors therefore concluded that this simple and cost-effective method may be useful for early PHPT screening, particularly in settings where PTH measurement is less accessible.

Collectively, the contributions to *Current Perspectives in the Diagnosis of Parathyroid Disease – Volume II* reflect a clear paradigm shift in the field. Diagnosis is no longer limited to biochemical confirmation or anatomical localization but increasingly incorporates integrative risk assessment, biological characterization, and technology-enhanced evaluation. These advances have the potential to refine patient selection, optimize therapeutic strategies, and improve clinical outcomes. Future research should prioritize the prospective validation of emerging biomarkers, standardization of advanced imaging techniques, and integration of artificial intelligence and digital modeling into clinical workflows. Through these efforts, innovations in parathyroid disease diagnostics can be effectively translated into precision care.

